# The Management of Postpartum Cardiorespiratory Failure in a Patient with COVID-19 and Sickle Cell Trait Requiring Extraorporeal Membrane Oxygenation Support and Airflight Transportation

**DOI:** 10.3390/jcm14010213

**Published:** 2025-01-02

**Authors:** Alexandre Pelouze, Sylvain Massias, Diae El Manser, Adrien Koeltz, Patricia Shri Balram Christophe, Mohamed Soualhi, Marc Licker

**Affiliations:** 1 Department of Cardiovascular & Thoracic Anaesthesia and Critical Care, University Hospital of Martinique, F-97200 Fort-de-France, Martinique, France; sylvain.massias@chu-martinique.fr (S.M.); diae.elmanser@chu-martinique.fr (D.E.M.); adrien.koeltz@chu-martinique.fr (A.K.); shri.balram@chu-martinique.fr (P.S.B.C.); mohamed.soualhi@chu-martinique.fr (M.S.); marc.licker@unige.ch (M.L.); 2 Faculty of Medicine, University of Geneva, CH-1206 Geneva, Switzerland

**Keywords:** cardiogenic shock, pregnancy, extracorporeal life support, sickle cell anemia, Afro-Caribbean ethnicity

## Abstract

Acute cardiovascular disorders are incriminated in up to 33% of maternal deaths, and the presence of sickle cell anemia (SCA) aggravates the risk of peripartum complications. Herein, we present a 24-year-old Caribbean woman with known SCA who developed a vaso-occlusive crisis at 36 weeks of gestation that required emergency Cesarean section. In the early postpartum period, she experienced fever with rapid onset of acute respiratory distress in the context of COVID-19 infection that required tracheal intubation and mechanical ventilatory support with broad-spectrum antibiotics and blood exchange transfusion. Shortly thereafter, transthoracic echocardiography documented severe biventricular dysfunction associated with raising levels of cardiac troponin and ECG signs of myocardial ischemia. Medical treatment with incremental dobutamine and noradrenaline infusion failed to improve cardiac output and blood gas exchange. After consultation with the regional cardiac center, a prompt decision was made to provide cardiac and respiratory support via implantation of femoral cannula and initiation of veno-arterial extracorporeal membrane oxygenation (ECMO, Cardiohelp^®^). Under stable ECMO, the patient was transferred by helicopter to a specialized cardiac center. There were no signs of ongoing hemolysis, and progressive recovery of the right and left ventricular function facilitated forward blood flow through the aortic valve. Three days after implantation, ECMO was weaned, and the cannula were removed. One day later, the patient’s chest X-rays showed partial resolution of lung edema. The patient was successfully extubated, and non-invasive ventilation with pulmonary rehabilitation was initiated to speed up her functional recovery.

## 1. Introduction

Over the last two decades, mortality related to obstetric complications, namely hemorrhage and infection, has steadily declined, whereas deaths from non-obstetric pathologies, particularly cardiac diseases, have remained stable despite surgical advances in correcting congenital heart disease, antenatal diagnosis of cardiac abnormalities, and optimization of medical treatments [1].

Overall, cardiac diseases complicate 1 to 4% of pregnancies, with higher rates observed in low- and middle-income countries [2]. In Western countries, up to 33% of maternal deaths are attributed to cardiovascular disorders, namely decompensation of pre-existing cardiac disorders, uncontrolled hypertension, pulmonary embolism, stroke, aortic dissection, or peripartum cardiomyopathy (PPCM) [2]. Among women of Afro-Caribbean descent, sickle cell anemia (SCA)—an autosomal recessive blood disorder—increases the risk of maternal and fetal death as a result of anemia, thrombosis, and acute vaso-occlusive crisis that develops in various organs, including the heart and lungs [3].

Cardiogenic shock (CS) is the most extreme cardiovascular condition during the peripartum period. Occasionally, mechanical circulatory support (MCS) with an intra-aortic balloon pump, ventricular assist device, or veno-arterial extracorporeal membrane oxygenation (VA-ECMO) is required as a bridge to functional recovery or cardiac transplantation [4]. The technical equipment and high expertise of ECMO necessitate specialized tertiary critical care centers with retrieval teams to implant on-site ECMO (”primary mission”) and transfer the supported and stabilized patient on ECMO to the tertiary care center for further treatment (”secondary mission”). Such air-medical inter-hospital transfers are complex and require close collaboration between critical care physicians, cardiac surgeons, and emergency rescue teams for proper patient evaluation, planning interventions, and safe transportation within the shortest time [5].

Herein, we describe the case of a young woman with SCA who presented acute cardiorespiratory distress following a cesarean section that required VA-ECMO support and transfer by helicopter to a referral cardiac intensive care unit (ICU).

## 2. Case Report

A 24-old year Caribbean woman, working full-time as a hairdresser, with a history of SCA and current pregnancy (G2P1; 36 weeks) was admitted to the University Hospital of Guadeloupe in Pointe à Pitre for acute pain in her back and legs. At the age of five, she was diagnosed with sickle cell trait (SCT) clinically expressed by recurrent acute chest syndromes and vaso-occlusive crisis requiring ambulatory treatment by blood exchange transfusion as prescribed by her hematologist. Shortly after admission, she developed a fever with normal vital parameters and no signs of fetal distress. Laboratory testing revealed elevated cardiac troponin-T ([cTp-T], 1.450 ng/mL), low hemoglobin ([Hb] 9.0 g/dL with a mean corpuscular volume of 92 fL), and 9% of reticulocytes), 30% of the variant Hb S, and low haptoglobin level (52 mcg/mL); a peripheral blood smear demonstrated severe anisopoikilocytes with large numbers of elongated sickle cells. The transthoracic echocardiogram (TTE) showed a normal functioning heart. Clinical and biological evidence of acute red cell sickling prompted the decision for an emergent Cesarean section under general anesthesia that was successfully completed with minimal blood loss (400 mL) and delivery of a healthy boy (Apgar score of 8 at 1 min and 9 at 5 min).

Shortly after surgery, she presented with shortness of breath associated with diffuse bilateral pulmonary crackles and oxygen desaturation (oxygen pulsed saturation [SpO_2_] of 92% on room air), requiring admission to the ICU and the initiation of non-invasive ventilation with high levels of fractional inspired oxygen (FIO_2_). Fluorescence-based quantitative reverse transcriptase–polymerase chain reaction (RT-PCR) of a throat swab tested positive for coronavirus disease 19 (COVID-19), while a multiplex PCR respiratory panel (Biofire^®^ FilmArray^®^, Biomerieux, Craponne, France) was negative for other infectious agents. Chest computed tomography scan demonstrated bilateral areas of ground-glass opacities with no sign of thromboembolism, which supported the diagnosis of severe acute respiratory syndrome coronavirus 2 (SARS-CoV-2). Due to severe hypoxemia (arterial oxygen partial pressure [PaO_2_] of 55 mmHg with 50% FIO_2_), orotracheal intubation was carried out, and mechanical ventilation was initiated under full sedation and muscle paralysis. Empiric antibiotic therapy was started (ceftriaxone and amikacin), and exchange red blood cell transfusion was conducted with three units of packed red blood cells. Although oxygenation improved (PaO_2_/FIO_2_ ratio of 150), the hemodynamic condition deteriorated progressively (mean arterial pressure of 55 mmHg, heart rate 120 beats/min), cTp-T further increased (8.450 ng/mL), and the ECG showed peaked T waves with slight ST segment depression. The TTE demonstrated severe global hypokinesia with a left ventricular ejection fraction (LVEF) of 15%, a tricuspid annular peak systolic excursion (TAPSE) of 8 mm, velocity time integral in the LV outflow tract of 7 cm, and slight enlargement of the cardiac cavities. The administration of dobutamine (5–10 mcg/kg/min) and norepinephrine (tartrate, 0.3–0.6 mcg/kg/min) did not produce favorable clinical and echocardiographic changes, while serum creatinine and arterial lactate levels further increased (151 mg/dL and 3.5 mmol/L, respectively), central venous oxygen saturation (ScvO_2_) dropped from 70% to 61%, and veno-arterial difference in partial pressure of carbon dioxide (CO_2_ gap) widened from 6 to 12 mmHg. This advanced stage of CS was discussed with cardiac surgeons and ICU colleagues from the University Hospital of Martinique (UHM), and the decision was made to provide urgent mechanical cardiopulmonary support. Two hours later, the ECMO mobile team left the UHM, arrived at the ICU in Pointe à Pitre, and inserted heparin-bounded cannulas in the right common femoral artery and vein using the Seldinger technique in this critically ill woman. A catheter was placed in the right superficial femoral artery to preserve distal limb perfusion, and unfractionated heparin infusion was started to target an activated clotting time between 150 s and 180 s. The Cardiohelp^®^ pump (Maquet, Rastatt, Germany) was set at a flow rate of 3.5 L/min with a sweep gas flow of 3.0 L/min (FIO_2_ of 1.0), while tidal volume on the ventilator decreased from 7 to 4 mL/kg of the ideal body weight, and positive end-expiratory pressure (PEEP) increased from 6 to 9 cm H_2_O with a FIO_2_ at 0.6.

**Figure 1:**
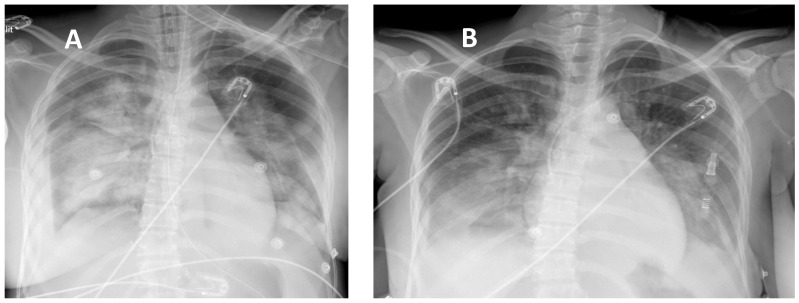
(**A**) Chest X-rays showing persistent lung edema with bilateral infiltrates under mechanical ventilation and extracorporeal membrane oxygenation; (**B**) chest X-rays showing partial regression of lung edema and improved aeration on spontaneous ventilation after tracheal extubation.

After 1 h of cardiopulmonary stabilization, the patient was transferred by helicopter to the cardiac ICU in Fort-de-France (195 km, 48 min) under protective mechanical ventilation and VA-ECMO support. After admission, chest X-rays showed persistent bilateral infiltrates with air bronchogram (Figure 1A); dobutamine infusion was interrupted, and norepinephrine was reduced to 0.2 mcg/kg/min, while the ECG ischemic changes disappeared with no indication to vent the left cavities given appropriate pulsed arterial pressure (>35 mmHg) and no signs of pulmonary congestion as documented by lung ultrasound examination. Two days after ECMO implantation, TTE showed partial recovery of both right and left ventricular function, and the flow rate of the ECMO was reduced to 2 L/min to promote left ventricular contraction and forward blood flow through the aortic valve. Blood sampling from the right radial artery showed adequate carbon dioxide removal and oxygen saturation exceeding 100 mmHg while decreasing both ECMO-FIO_2_ and sweep gas flow (from 1.0 to 0.5 and from 3.0 to 1.5 L/min) with similar protective ventilatory settings.

**Figure 2:**
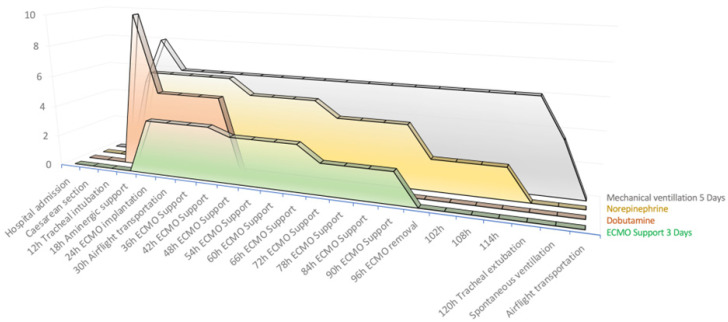
Timeline of key events (Cesarian section, respiratory failure and heart failure) and therapeutic interventions from hospital admission to weaning extracorporeal membrane oxygenation (ECMO) and mechanical ventilation followed by airflight transportation back to the referaal hospital.

Biological signs of inflammation regressed, and cultures from both blood and broncho-alveolar lavage fluids were all negative, allowing for the discontinuation of antibiotics. There were no signs of ongoing hemolysis, and blood hemoglobin levels remained stable (7.5 g/dL). On the 3rd day after implantation, the pump flow rate was gradually reduced, and after a successful ECMO weaning trial, the femoral cannulas were removed. On the 4th day, the trachea was extubated as the patient was able to breathe spontaneously with low levels of inspiratory pressure support and 30% FIO_2_. Two days later, chest X-rays showed partial resolution of lung edema with residual areas of consolidation (Figure 1B), while TTE demonstrated improvement in LVEF and TAPSE (55% and 15 mm, respectively) with normalization of the LV end-diastolic dimension (40 mm). The patient was discharged from the cardiac ICU in Martinique and transferred back to the obstetric department in Guadeloupe on oral medical treatment (aspirin 75 mg, bisoprolol 2.5 mg, spironolactone 25 mg, bromocriptine 2.5 mg, and rivaroxaban 10 mg). Before returning home, the patient started a 2-week rehabilitation program. After 3 months, the patient resumed her professional activities at 50% as well as her daily home tasks. Figure 2 shows the timeline of key intervention events during the treatment from her admission to her discharge.

Data were taken from Table 1 with adjusted values in order to be spotted on the same chart as follows:

1. Mechanical ventilation FiO_2_ ×10;
2. ECMO Pump flox in L/min;
3. Norepinephrine in mcg/kg/min ×10;
4. Dobutamine mcg/kg/min ×1.

## 3. Discussion

This is the first report of a patient with SCT who developed acute cardiorespiratory failure following cesarean section requiring emergent mechanical ventilation, VA-ECMO support, and airflight transportation. We hypothesize that SARS-Cov-2 infection in late pregnancy was associated with a sickle cell crisis involving the coronary microvasculature that led to acute heart failure.

### 3.1. CS in Pregnant Women and Treatment with VA-ECMO

Owing to major physiological changes associated with pregnancy (i.e., increased blood volume and cardiac output, prothrombotic status), acute heart failure with CS is more likely to occur in women with pre-existing heart diseases, preeclampsia, various forms of cardiomyopathies (i.e., familial, arrhythmogenic, and left ventricular noncompaction) or as a result of amniotic fluid embolism or spontaneous coronary dissection [6]. When the aforementioned heart disorders are excluded, the diagnosis of peripartum cardiomyopathy (PPCM) is retained with its multifactorial etiologies, including genetic mutations, viral myocarditis, nutritional deficiencies, and autoimmune disorders [7]. During pregnancy, PPCM is the most common cause of acute heart failure, with a higher incidence in sub-Saharan Africa and Caribbean islands than in Western countries (1 in 300 births versus 1 in 1000–4000) [8].

Using the Society of Cardiovascular Angiographic and Interventions [SCAI] classification of the severity of CS, the in-hospital mortality rate increases from 16% to 62% across stages C, D, and E [9]. Analysis of the US Nationwide Readmission database from 2010 to 2020 (N = 39,790,772 deliveries) showed that CS occurred in 776 parturients (40% PPCM), with 18.8% mortality (compared with 0.02% without CS) [9,10]. Over the last few decades, a three-fold increase in CS incidence has been observed, associated with a 25% increased incidence of pre-existing heart diseases among pregnant women (e.g., congenital cardiac abnormalities and acquired valvular diseases).

In the 2022 guidelines from the American Heart Association/American College of Cardiology/Heart Failure Society of America, treatment with any MCS devices (intra-aortic balloon pump (IABP), left ventricular assist device (LVAD), or VA ECMO is advocated in patients with advanced heart failure (class IIa) [11]. These recommendations are largely based on the analysis of patients with heart failure following cardiac surgery, acute myocardial infarction, cardiac arrest, myocarditis, or sepsis. Despite improvement in circulatory flow under MCS, short-term outcomes are relatively poor. A meta-analysis of 32 cohort studies, including patients with CS (N = 12,756), reported 62% in-hospital mortality along with high incidences of major complications, namely acute renal failure (50%), bleeding (48.5%), multiple organ dysfunction (24.4%), or stroke (12.5%) [12].

Data regarding pregnant women with CS are relatively scarce. Regardless of the type of device, better outcomes have been reported after MCS application, which could be attributed to their younger age, fewer comorbidities, and less severe associated organ dysfunction [13,14,15]. In our patient, the presence of bi-ventricular failure coupled with acute respiratory failure ruled out the indication for IABP or LVAD, leaving VA ECMO as the best treatment option to support the failing heart and lungs. In the last analysis of the US National Inpatient Sample from 2016 to 2020 (N = 19,524,846), VA-ECMO was applied in 4455 women with CS, including 125 (2.8%) pregnant women who demonstrated better survival (71%) and a lower incidence of acute renal failure and sepsis (6.5%) [16]. Likewise, in the last summary of the international registry of the Extracorporeal Life Support Organization (ELSO; N = 56,210), 277 pregnant women were treated with VA-ECMO (N = 128), VV ECMO (N = 113), or a hybrid mode (N = 36) for refractory CS, thromboembolism, amniotic fluid embolism, and respiratory failure [15]. The survival rate of 70% in pregnant women (65.6% for VA ECMO, 74.1% for VV-ECMO) was higher than in non-pregnant adults with respiratory or cardiac failure (59% and 42%, respectively) [17]. In our patient, the SAVE (Survival After Veno-arterial ECMO) score that was developed from the ELSO registry (N = 3846) indicated a mortality risk of 25% [18].

So far, given the limited number of pregnant women with CS, the optimal use of MCS for this sub-population cannot be properly addressed. Therefore, dedicated goal-directed medical treatment protocols and techniques using the new generation of canula and pumps should be applied to reverse circulatory failure and reduce the occurrence of adverse events in women with peripartum CS.

### 3.2. Sickle Cell Anemia

SCA arises from a missense mutation in the HBB gene encoding the β-globin subunit of hemoglobin. Accordingly, the substitution of glutamic acid (hydrophilic) for valine (hydrophobic) in the 6th position of the β-chain produces a less soluble form of hemoglobin (HbS) than the normal adult form of Hb (HbA) [19]. Under regional hypoxic conditions, red blood cells undergo morphological changes due to HbS polymerization, resulting in shortened life span of red cells, increased blood viscosity, arteriolar vasoconstriction, and endothelial damage that promote low microvascular flow and vascular occlusion [20].

The homozygous (Hb-SS) and the heterozygotous forms (Hb-SS and HbSA or SCT, respectively) of SCA affect 1 in 400 to 600 and 1 in 12 to 14 Afro-American and Caribbean people, respectively [21]. In most cases, SCT disease is clinically milder than Hb-SS disease but is also associated with reduced survival and poor quality of life owing to higher prevalences of congestive heart failure, stroke, thromboembolism, kidney disease, leg ulcers, and pulmonary lesions [22]. Contrasting with the general asymptomatic presentation of SCT carriers, recent clinical studies and case series suggest that these SCT carriers may also suffer from severe vaso-occlusive crises triggered by stressful events that require hospitalizations and blood exchange therapy, as witnessed in the present case. From a population-based cohort of 8.8 million births, 1210 pregnant women exhibited acute crisis; 89.8% of them were diagnosed with Hb-SS and 3.14% with SCT [23]. The occurrence of vaso-occlusive events was associated with a higher risk of maternal mortality (odds ratio (OR) of 15.8 and 95% confidence interval (CI) of 1.8 to 135.6) and cardiomyopathy (OR of 13.8 and 95% CI of 1.6 to 122.9) [23]. To manage these pregnant women with SCA, the British Society for hematology guidelines recommend the following measures: a multidisciplinary antenatal care team involving obstetricians, hematologists, and cardiologists (grade 1C); discontinuation of hydroxycarbamide (2C); and iron chelators (grade 1B); antibiotic prophylaxis (grade 1A); updated vaccinations (i.e., flu, COVID; grade 1B); supplementation with folic acid daily (5 mg; grade 1A), and iron if there is laboratory evidence of deficiency (grade 1A); aspirin daily starting from 12 weeks of gestation (75–150 mg; grade 1B); and exchange blood transfusion if worsening anemia or complications (grade 1B) [24].

In the current case, the pregnant woman failed to follow these recommendations, except for blood transfusion. At hospital admission, the clinical expression of the vaso-occlusive crisis was confirmed by laboratory markers of hemolysis [25], and CS developed shortly after the onset of SARV-CoV-2. Hypoxia-induced red blood cell sickling within the coronary microvasculature likely promoted diffuse myocardial ischemia as reflected by concomitant elevation of cTp blood levels and appearance of peaked T wave with ST depression at the ECG. Two cohort studies confirmed that the elevation of cTp correlates with the burden of hemolysis, suggesting that myocardial ischemia and cardiac stunning may result from the obstructed flow within the coronary microvasculature and endothelial lesions during sickle cell crisis [26,27].

From 1987 to 2022, 25 cases of ECMO treatment were described in patients with SCA (20 VV ECMO and 5 VA ECMO) who experienced acute chest syndrome with refractory hypoxia and/or cardiogenic shock [28]. After an average ECMO run of 14 days, the 30-day mortality rate was 16%. A single case of a 25-week pregnant woman with SCA was reported. Sickling-induced ARDS was successfully managed with red blood cell exchange transfusion, mechanical ventilation, and VV-ECMO support [29]. These critically ill patients recovering from cardiogenic shock with or without respiratory failure should benefit from rehabilitation program to maximize their functional recovery [30]. Multicomponent programs combining exercise, breathing training, psychological counseling, and nutritional advice are effective in reducing fatigue and dyspnea on exercise while improving muscle strength and emotional balance [31].

### 3.3. Airflight Transportation of High-Risk Cardiac Patients in French West Indies

As reported from the Stockholm experience and the Japanese nationwide database, complications associated with medical air transportation should be anticipated and are categorized as related to the patient’s condition (i.e., bleeding, decannulation, thromboembolism, limb ischemia, and low flow), staffing (i.e., experience and communication), equipment (i.e., electrical power or gas supply failure), and vehicle and environment (i.e., extreme temperature and decompression accident) [32,33]. Equipment for inter-hospital transport on ECMO should be checked by well-trained and experienced healthcare professionals. The ECMO chart should contain circuit tubing, bladder box and venous outflow reservoir, pump, membrane oxygenator, inline blood gas monitor, cardiorespiratory monitor, heat exchanger, arterial and thrombus filter, and Doppler flowmeter, mechanical ventilator, water heater/pump, and gas tanks.

The department of cardiothoracic surgery at the UHM in Fort de France provides all types of cardiac interventions—except transplantations—for approximately 1.4 million people living in the French West Indies territories (Martinique, Guadeloupe, and St Martin) as well as the French Guiana and some neighboring islands (Dominica and Saint Lucia). As these territories are hundreds of kilometers apart, aeromedical transport for emergent cases needs to be rapidly organized by helicopter or by military airplane, with close communication between healthcare providers from these remote locations and the referral cardiac center in Martinique. Since 2010, the department of cardiac surgery has set up an ECMO mobile team to perform on-site VA ECMO implantations and repatriation of these critically ill patients to the cardiac ICU at the UHM. All cases are discussed with cardiac surgeons and ICU physicians who provide advice for the optimization of pharmacological treatment and to validate the decision of VA-ECMO implantation [34,35]. Early referral of high-risk pregnant women with severe heart diseases may prevent delays and adverse outcomes. Whenever possible, these high-risk women are transferred to the UHM, where delivery is planned under advanced hemodynamic monitoring and eventual MCS. Over the last 5 years, 35 VA-ECMOs have been implanted in patients with heart failure in Pointe à Pitre (Guadeloupe) and in Cayenne (French Guiana). After hemodynamic and respiratory stabilization, these patients have been transferred with airborne mobile ICU to the cardiac ICU in Fort de France.

Such a “flying bridge” allows critically ill patients with CS to be transferred from a remote center to a specialized cardiac center with/without MCS until functional recovery or further evaluation for heart transplantation or long-term VAD. Given the limited access to MCS in many overseas territories and the long distances between hospital centers, there is a need to develop regional networks with dedicated ECMO centers and to implement standardized protocols for safe transportation [36].

## 4. Conclusions

This is the first case report of a pregnant woman with symptomatic SCT who developed SARS CoV-2 with refractory CS after an uneventful Cesarean section. Her acute cardiopulmonary failure was successfully managed over a short period of mechanical ventilation and VA ECMO.

The current literature concerning pregnant patients with CS and treated with VA ECMO is limited to anecdotal case reports and a few cohorts (ELSO registry, US databases). Given their younger age and lesser comorbid condition, the prognosis of peripartum CS is better than in non-pregnant individuals. Analysis of large databases, including such pregnant patients, is needed to determine the outcome after VA-ECMO support and to develop dedicated guidelines in this special subgroup of patients with CS.

## Figures and Tables

**Table 1:** Summary of clinical events, physiological markers, and interventions from hospital admission and Cesarean section (Cs) followed by cardiopulmonary failure and recovery after ECMO implementation.

Parameter Interventions	Hospital Admission	12 h Post-Cs	18 h Post-Cs	24 h Post-Cs	48 h Post-Cs	72 h Post-Cs	96 h Post-Cs	120 h Post-Cs
BP, mmHg	105/60	95/55	88/54	65/55	63/52	89/67	93/70	108/72
HR, b/min	88	105	120	132	125	105	95	98
SpO_2_/FIO_2_	0.99/0.21	0.90/0.50	0.88/0.8	0.99/0.60	0.99/0.30	0.97/0.5	0.99	0.96/0.35
cTp-T, ng/mL	1.450	-	8.450	-	2.340	1.910	0.825	0.610
ECG	Normal	Sinus tachycardia	Peak T, ST depression	Peak T, ST depression	Normal	Normal	Normal	Normal
TTE	Normal	-	LVEF 15%, TAPSE 8, LVOT-VTI 7	Canula in IVC unloaded RV	LVEF 40% TAPSE 10 LVOT-VTI 10	LVEF 45% TAPSE 12 LVOT-VTI 11	-	LVEF 55% TAPSE 15 LVOT-VTI 14
Ventilatory Mode	Spontaneous Ventilation	Non-Invasive Ventilation	Mechanical Ventilation	Mechanical Ventilation	Mechanical Ventilation	Mechanical Ventilation	Mechanical Ventilation	Spontaneous Ventilation
Ventilatory Settings	Room air	FIO_2_ 0.5, PEEP 6	FIO_2_ 0.8 PEEP 6 Vt 7	FIO_2_ 0.6 PEEP 9 Vt 4	FIO_2_ 0.6 PEEP 9 Vt 4	FIO_2_ 0.6 PEEP 9 Vt 4	FIO_2_ 0.6 PEEP 9 Vt 4	FIO_2_ 0.35
Medical treatment		Ceftriaxome Amikacin 3 RBC units	Dobu 10 NE 0.6	Dobu 5 NE 0.6	Dobu 0 NE 0.5	Dobu 0 NE 0.4	Dobu 0 NE 0.2	Dobu stop NE stop
ECMO Settings	Pump flow Sweep gas FIO_2_	-	-	3.5 L/min 3.0 L/min 1.0	3.0 L/min 2.5 L/min 0.8	2.0 L/min 1.5 L/min 0.5	Stop ECMO	-

BP, blood pressure; cTp-T, cardiac troponin-T; Dobu, dobutamine; ECG, electrocardiogram; ECMO, extracorporeal membrane oxygenation; FIO_2_, fractional inspiratory oxygen; HR, heart rate; LVEF, left ventricular ejection fraction; LVOT-VTI, left ventricular outflow tract–velocity time interval (in cm); NE, norepinephrine; PEEP, positive end-expiratory pressure; SpO_2_, pulsed oxygen saturation; TAPSE, tricuspid annular plane systolic excursion (in mm); TTE, transthoracic echocardiography; Vt, tidal volume (in ml/predicted body weight).
